# TGFB1 polymorphisms and TGF‐β1 plasma levels identify gastric adenocarcinoma patients with lower survival rate and disseminated disease

**DOI:** 10.1111/jcmm.16131

**Published:** 2020-12-04

**Authors:** Ignacio Juarez, Alberto Gutierrez, Christian Vaquero‐Yuste, Elisa M. Molanes‐López, Adela López, Inmaculada Lasa, Remedios Gómez, José Manuel Martin‐Villa

**Affiliations:** ^1^ Department of Immunology, Ophthalmology and ENT Facultad de Medicina Universidad Complutense de Madrid (UCM) Madrid Spain; ^2^ Hospital Universitario Príncipe de Asturias Madrid Spain; ^3^ Department of Statistics and Operations Research Facultad de Medicina Universidad Complutense de Madrid (UCM) Madrid Spain; ^4^ Instituto de Investigación Sanitaria Gregorio Marañón (IiSGM) Madrid Spain

**Keywords:** gastric cancer, metastasis, SNPs, TGF‐beta

## Abstract

TGF‐β1 is involved in tumour growth. Four TGFB1 SNPs and TGF‐β1 production by stimulated PBMC were determined in seventy‐eight gastric adenocarcinoma patients. In addition, TGF‐β1 levels were measured in the plasma of further thirty patients. rs1800471‐G/C genotype was prevalent in patients (20.7%) compared to controls (8.4%), as it also was the rs1800468 SNP‐G/A genotype in stage IV patients (20.7%) compared to stage I, II and III patients, combined (10.3%). Conversely, the T/T rs1800469 SNP‐T/T genotype was absent in the former group and present in 19.0% in the latter. Furthermore, the rs1800469‐C/rs1800470‐T (CT) haplotype was found in 15.0% of stage IV patients as compared to 3.0% of the remaining patients (3.0%) and also identifies patients with worse five‐year life expectancy (*P* = .03). TGF‐β1 synthesis by stimulated PBMCs was significantly lower in patients with the risk SNPs or haplotype, compared to the alternative genotype. Finally, TGF‐β1 plasma levels were lower in patients with worse life expectancy. Analysis of TGFB1 SNPs and measurement of plasma TGF‐β1 levels serves to identify patients at risk of developing a more aggressive disease.

## 1 INTRODUCTION

Gastric cancer is the fifth most common type of malignancy in the world (over one million cases in 2018) and the third leading cause of cancer‐related deaths worldwide (783 000 deaths in 2018).^1^ In Spain, expected figures are 7963 estimated new cases in 2020 and 5809 deaths.

Clinical symptoms of gastric cancer appear late in the evolution of the disease, and this may limit the early detection of the pathology and the patients’ therapeutic options; thus, upon diagnosis of gastric cancer, prognosis is poor (5‐year survival rate below 29%). The need for early diagnosis and prognosis criteria has led recent research to focus on the investigation of novel biomarkers which could help identify patients at risk of developing more threatening forms of gastric cancer.^2^, ^3^, ^4^, ^5^ The identification of patients with poor prognosis allows the adoption of more radical therapeutic or surgical approaches upon diagnosis.

Gastric adenocarcinoma is the main type of gastric tumours and accounts for more than 90% of cases. Complex genetic and environmental factors interact and, together, lead to its initiation and progression. These factors, along with the high rate of somatic mutations,^6^ can play an important role in the malignancy and low survival rate of gastric cancer.

The tumour microenvironment is involved in the development and evolution of this pathology. The immune system, as the principal mediator of the inflammatory response taking place at the gastric epithelium in response to infectious or other agents, may play a dual role in the progression of the malignancy, whether fostering or limiting the tumour growth and dissemination. Inflammation and DNA damage are mutually related, as each one can be the initiator of the other, leading to mutations, hyperplasia and, finally, neoplasia.^7^ However, immune cells also play a protective role in the cancer immunoediting hypothesis, whereby the immune system can recognize and eliminate tumours in the initial stages of the pathology.

TGF‐β1 is a cytokine that plays a dual role in gastric cancer, either promoting cancer development, acting as a factor that inhibits immunosurveillance and promotes epithelial‐mesenchymal transition (EMT) and metastases or, alternatively, suppressing tumour growth by directly inhibiting cell cycle progression, leading to growth arrest and activation of apoptotic pathways.^8^, ^9^ TGF‐β1 can be synthetized by healthy epithelia and, in the tumour microenvironment, by immune and tumoural cells. Studies focused on TGF‐β1 at the gene or protein level were carried out by several authors in other types of cancer, but they are scarce in gastric cancer.

The gene that codifies this cytokine, TGFB1, includes several single nucleotide polymorphisms (SNPs). In this study, we focused on four of these SNPs: rs1800468, rs1800469 (both located in the promoter), rs1800470 and rs1800971 (both in the exon 1, that codifies for the signal peptide of the protein). These polymorphisms can modify the expression of the protein, either affecting the joining of transcription factors to the promoter region of the gene^10^, ^11^ or modifying the effective secretion of the molecule to the extracellular medium.^12^, ^13^, ^14^ In fact, genetic polymorphisms of the TGFB1 gene were already associated with increased or reduced risk of development and evolution of other types of pathologies mainly related to the immune response and inflammation processes.^15^, ^16^, ^17^, ^18^, ^19^

Thus, we studied these polymorphic variants in a population of patients with gastric adenocarcinoma in different clinical stages of development (TNM‐7th edition 2009 UICC/AJCC),^20^ as well as in a population of non‐affected controls. Moreover, we wished to assess the effect of the different SNPs on the levels of the TGF‐β1 produced by PBMC. Finally, we measured TGF‐β1 plasma levels to determine whether we could identify patients with worse prognostic.

Results achieved would contribute to establishing TGFB1 gene SNPs as new possible diagnostic and prognostic biomarkers as well as clarifying the actual role of this cytokine in gastric cancer.

## 2 PATIENTS, MATERIALS AND METHODS

### 2.1 Patients

One‐hundred and eight patients with gastric adenocarcinoma undergoing surgery (at Hospital Universitario Príncipe de Asturias, Alcalá de Henares, Madrid, Spain) were involved. This cohort was split as follows: seventy‐eight patients were subjected to DNA extraction and subsequent TGFB1 SNPs analysis and measurement of TGF‐β1 production by purified PBMCs, and thirty patients were used to measure plasma TGF‐β1 levels.

Patients were classified according to TNM‐7th edition 2009 (UICC/AJCC) criteria and grouped in stages I (early cancer), II, III (locally advanced disease) and IV (disseminated disease) (Table 1).

**TABLE 1:** Patients’ characteristics according to age, sex, stage of the disease, treatment and survival. Seventy‐eight patients were included in the TGFB1 SNPs study and thirty different patients to measure the levels of TGF‐β1 in plasma

	Patient characteristics				
	Total N. (%)	SNP analysis		Plasma TGF‐β1	
		78	(100)	30	(100)
Age (y)	Median (range)	67	(37‐89)	65	(37‐84)
Sex, N. (%)	Male	50	(64)	16	(53)
	Female	28	(36)	14	(47)
Stage, N. (%)	I	21	(27)	10	(33)
	II	18	(23)	13	(43)
	III	19	(24)	0	(0)
	IV	20	(26)	7	(23)
Treatment^a^, N. (%)	Surgery	78	(100)	30	(100)
	Chemotherapy	78	(100)	30	(100)
Localization, N. (%)	Fundus	12	(15)	2	(7)
	Antrum	31	(40)	15	(50)
	Body	29	(37)	13	(43)
	Cardia	6	(8)	0	(0)
Type, N. (%)	Intestinal	47	(60)	16	(53)
	Non‐intestinal	31	(40)	14	(47)
Overall survival, N. (%)	<5 y	57	(73)	6	(20)
	>5 y	21	(27)	24	(80)

Note

All patients were treated after surgery and sample obtainment.

^a^ Cisplatin/Oxiplatin + 5‐FU + anthracycline.

### 2.2 Control patients

Two sources of control individuals were used:

1. Fifty‐four non‐cancer individuals were included as a control group. DNA was obtained either from blood or saliva samples. Methods to isolate DNA are described in the corresponding section.
2. In addition, and to assess differences in the distribution of the SNPs between patients and controls in a larger cohort, a group of 106 individuals from the same location (Iberian population in Spain), genotyped for the TGFB1 polymorphisms previously mentioned and included in the 1000 Genomes Project database,^21^ were added.

### 2.3 Materials and methods

#### 2.3.1 Biological samples

##### Tissue

Upon surgery, a sample of gastric tumoural or distal tissue was obtained from each patient.

##### Blood

Blood was obtained from gastric cancer and controls in EDTA‐containing tubes, to isolate PBMC and extract DNA, and plasma samples.

##### DNA isolation

DNA from tissue samples and peripheral blood was carried out using the Nucleon BACC kit (GE Healthcare), following the manufacturer instructions. DNA from saliva was isolated with the kit Oragene DNA (DNAgenotek) and cleaned with the reagent prepIT L2P (DNAgenotek), following the manufacturer instructions.

### 2.4 Genetic studies

DNA samples were then employed for the genotyping of four SNPs of the TGFB1 gene: rs1800468, rs1800469, rs1800470 and rs1800471. To assess the results obtained, three different approaches were used in all patients tested. Genotyping data were considered consistent when at least two of the three methods used yielded concordant results.

#### 2.4.1 PCR‐RFLP

Primers and PCR conditions used for each SNP are shown in Table 2. DNA samples were amplified and resolved in 2% agarose gels and, after ensuring the correct amplification, PCR products of the SNPs rs1800468, rs1800469 and rs1800470 were further digested with the enzymes HpyCH4IV, Bsu36I and PstI‐HF (New England Biolabs), respectively.

**TABLE 2:** Primers and PCR conditions. rs1800468 and rs1800469, as well as rs1800470 and rs1800471, were analysed in the same PCR protocol because of their proximity

SNP	Primer Fwd (5'‐3')	Primer Rvs (5'‐3')	Cycles	Denaturation (TºC, time)	Annealing (TºC, time)	Elongation (TºC, time)
rs1800468 & rs1800469	GGCAGTTGGCGAGAACAGT	ACCCAGAACGGAAGGAGAGT	35	94°C 30 s	60°C 45 s	72°C 1 min
rs1800470 & rs1800471	ACCACACCAGCCCTGTTCGC	AGTAGCCACAGCAG CGGTAGCAGCTGC	33	94°C 30 s	66°C 1 min	72°C 1 min

#### 2.4.2 Taqman assay

Genotyping was also carried out by means of the allelic discrimination Taqman assay, using VIC‐ and FAM‐labelled probes. These tests are pre‐designed (Applied Biosystems) for each of the polymorphisms analysed in this study. Results were analysed with the Software Detection System v2.4 program (Applied Biosystems). 7900 HT Fast Real‐Time PCR System (Applied Biosystems) was used with the following PCR cycles: 95°C 10′; 40 cycles of 15 seconds at 95°C and 1′ at 60°C. The data were analysed with the SDS v2.4 program (Software Detection Systems, Applied Biosystems).

#### 2.4.3 DNA sequencing

DNA sequencing was performed with the PCR‐SSOP Luminex technique, and ambiguities were resolved by direct DNA sequencing.

### 2.5 Cytokine studies

#### 2.5.1 Peripheral blood mononuclear cell (PBMC) isolation and stimulation

PBMC were obtained by density gradient centrifugation using Ficoll Paque Plus (Sigma Aldrich). 200 000 cells per well were incubated with PMA (20 ng/mL) and Ionomycin (1 μmol/L) for 4 hours (37°C, 5% of CO_2_).

#### 2.5.2 ELISA

Assessment of TGF‐β1 protein produced by the stimulated PBMC, or assessment of plasma levels, was carried out with a TGF‐β1 ELISA kit (Enzo Life Sciences) following the manufacturer's instructions.

### 2.6 Statistical analysis

The data of the SNPs sequencing were analysed with the software SNPStats,^22^ This software allows to assess Hardy‐Weinberg Equilibrium (exact test), chi‐square test, OR estimation analysis of association between polymorphisms and disease applying logistic regression models, that consider the dominant, recessive and codominant models of inheritance. SNPstats also allows the analysis of linkage disequilibrium, using the D statistic and a correlation coefficient, and the analysis of haplotypes (EM algorithm). Kaplan‐Meier method was used to estimate the 5‐year survival function of patients with gastric cancer and different genetic factors. Multivariate Cox regression models were used to simultaneously assess the effect of genetic factors and other factors such as comorbidities, clinical features and demographic characteristics on 5‐year survival of patients with gastric cancer. For all the Cox regression fits, the individual and global Schoenfeld test indicated that none covariate in the model nor the model as a whole violate the Proportional Hazard assumption, meaning that the hazard ratio stays constant over time. Statistical analysis of the TGF‐β1 protein levels (*T* test) and Kaplan‐Meier survival curves were made with the GraphPad Prism 7.0 software. Additionally, Cox regression analysis was performed (R software) to analyse the effect in the survival rate of covariables and comorbidities of the patients. Cox Regression analysis was performed with the software R.

## 3 RESULTS

### 3.1 Genetic studies

#### 3.1.1 TGFB1 genetics in cancer susceptibility and progression

Hardy‐Weinberg equilibrium was confirmed for each SNP and group of individuals included in the study and combinations thereof. Also, linkage disequilibrium was assessed between the following pairs of SNPs: rs1800468‐rs1800469, rs1800468‐rs1800470, rs1800469‐rs1800470, rs1800469‐rs1800471 and rs1800470‐rs1800471 (data not shown).

##### Patients vs controls

Upon comparison with the control group, patients showed no significant differences in the frequency of the rs1800468, rs1800469 and rs1800470 polymorphisms (*P* > .05 in all instances, data not shown). However, when we compared the rs1800471 SNP in our cohort of patients with a genetically related control group from the 1000 Genomes Project database, a significant increase in the frequency of the G/C genotype in the group of patients (20.7%) was observed compared to the control group (8.4%, *P* = .027, OR 2.84) (Table 3A), revealing that this polymorphism is involved in the pathogenesis of gastric cancer.

**TABLE 3:** Polymorphisms were analysed according to all possible inheritance models for each SNP (but the SNPs rs1800468 and rs1800470, because of the absence of patients bearing the second homozygous genotype A/A and C/C, respectively).

	Inheritance Model	Genotype	Controls (1000 Genomes)		Gastric Adenocarcinoma		OR	95% Cl	*P*‐value
			n	%	n	%			
(A)									
rs1800471	N/Aᵃ	G/G	98	91.6	46	79.3	1	1	****.027****
		G/C	8	8.4	12	20.7	**2.84**	**(1.12‐7.22)**	

	Inheritance model	Genotype	Non‐Disseminated disease		Disseminated disease		OR	95% Cl	*P*‐value
			n	%	n	%			
(B)									
rs1800468	N/Aᵃ	G/G	52	89.7	14	70.0	1	‐	**.048**
		G/A	6	10.3	6	30.0	**3.71**	**(1.03‐13.40)**	
rs1800469	Codominant	C/C	25	43.1	10	50.0	1	‐	****.03****
		C/T	22	37.9	10	50.0	**1.15**	**(0.40‐3.32)**	
		T/T	11	19.0	0	***0.0***	**0**	**(0.00‐NA)**	
	Recessive	C/C‐C/T	47	81.0	20	50.0	1	‐	****.008****
		T/T	11	19.0	0	***0.0***	**0**	**(0.00‐NA)**	
	Dominant	C/C	25	43.1	10	50.0	1	‐	.62
		C/T‐T/T	33	56.9	10	50.0	0.77	(0.27‐2.16)	
rs1800470	Codominant	T/T	17	37.0	4	28.6	1		.57
		T/C	19	41.3	8	57.1	1.79	(0.45‐7.05)	
		C/C	10	21.7	2	14.3	0.85	(0.13‐5.61)	
	Dominant	T/T	17	37.0	4	28.6	1	‐	.56
		T/C‐C/C	29	63.0	10	71.4	1.47	(0.39‐5.50)	
	Recessive	T/T‐T/C	36	78.3	12	85.7	1	‐	.52
		C/C	10	21.7	2	14.3	0.59	(0.11‐3.14)	
rs1800471	N/Aᵃ	G/G	37	84.1	9	64.3	1	‐	.12
		G/C	7	15.9	5	35.7	2.99	(0.75‐11.83)	

ᵃ No inheritance model because of the lack of homozygous genotypes (A/A in rs1800468 and C/C in rs1800471).

Significant results are highlighted in bold.

Comparison with the fifty‐four healthy donors’ allelic distribution did not showed significant differences, maybe because of the low number of samples in study.

##### Inter‐patient comparisons (early stages vs disseminated disease)

Differences were also found in two of the SNPs studied (rs1800468 and rs1800469) when comparisons were carried out if the group of patients was broken down according to the severity of the disease (TNM staging). Thus, 30.0% of stage IV patients (disseminated disease) possess the G/A genotype of the rs1800468 SNP as compared to 10.3% of stage I, II and III patients, combined (*P* = .048, OR 3.71). Likewise, the T/T genotype of the rs1800469 SNP was absent in the stage IV group and present in 19.0% of the combined group of patients, with a significant change in the proportions of the genotypes either by a codominant (*P* = .03) or recessive model of inheritance (*P* = .008) (Table 3B).

**FIGURE 1:**
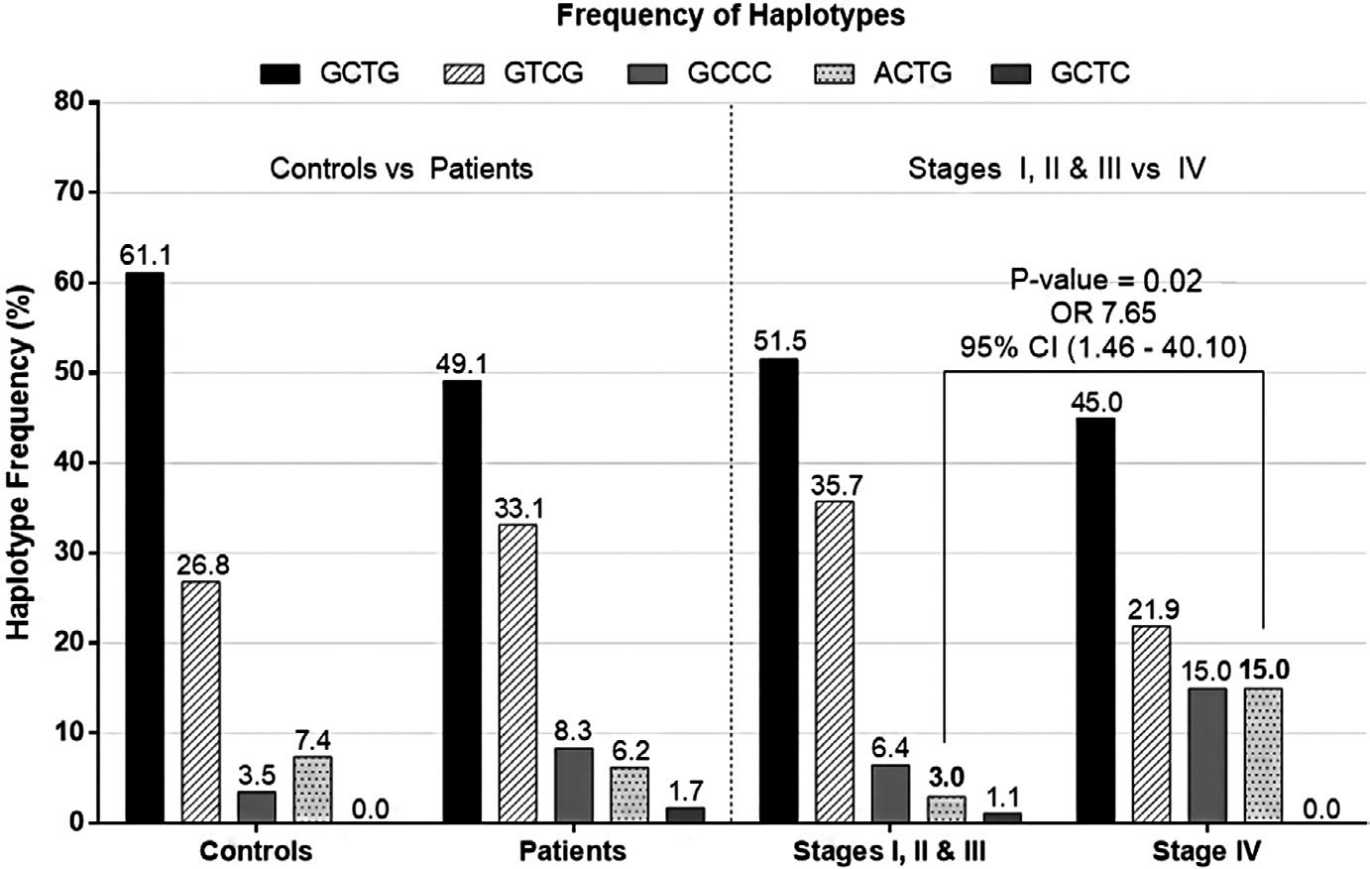
TGFB1 extended haplotypes. Controls, patients and subgroups of patients, according to the presence or absence of distant metastasis. Out of all the haplotypes studied, the ACTG haplotype was highly increased in the group of patients with metastasis (15.0%) compared to non‐metastatic ones (3.0%, *P* = .02; OR 7.65). This haplotype is linked to lower TGFB1 promoter activity, and, in fact, our results showed that patients with this haplotype produced less TGF‐β1 (see text)

We then analysed the frequency of the different extended haplotypes formed by the combination of the SNPs studied. The ACTG haplotype (of the rs1800468, rs1800469, rs1800470 and rs1800471 SNPs, respectively) was present in 15.0% of the stage IV patients as compared to 3.0% of stage I, II and III patients (*P* = .02, OR 7.65, Figure 1).

Because of the low number of patients bearing the rs1800468‐G/A genotype and, thus, the ACTG haplotype, we decided to use only the rs1800469 and rs1800470 SNPs to conform the risk haplotype rs1800469‐C/rs1800470‐T (CT) to make subsequent statistics.

##### TGFB1 polymorphism and survival curves

**FIGURE 2:**
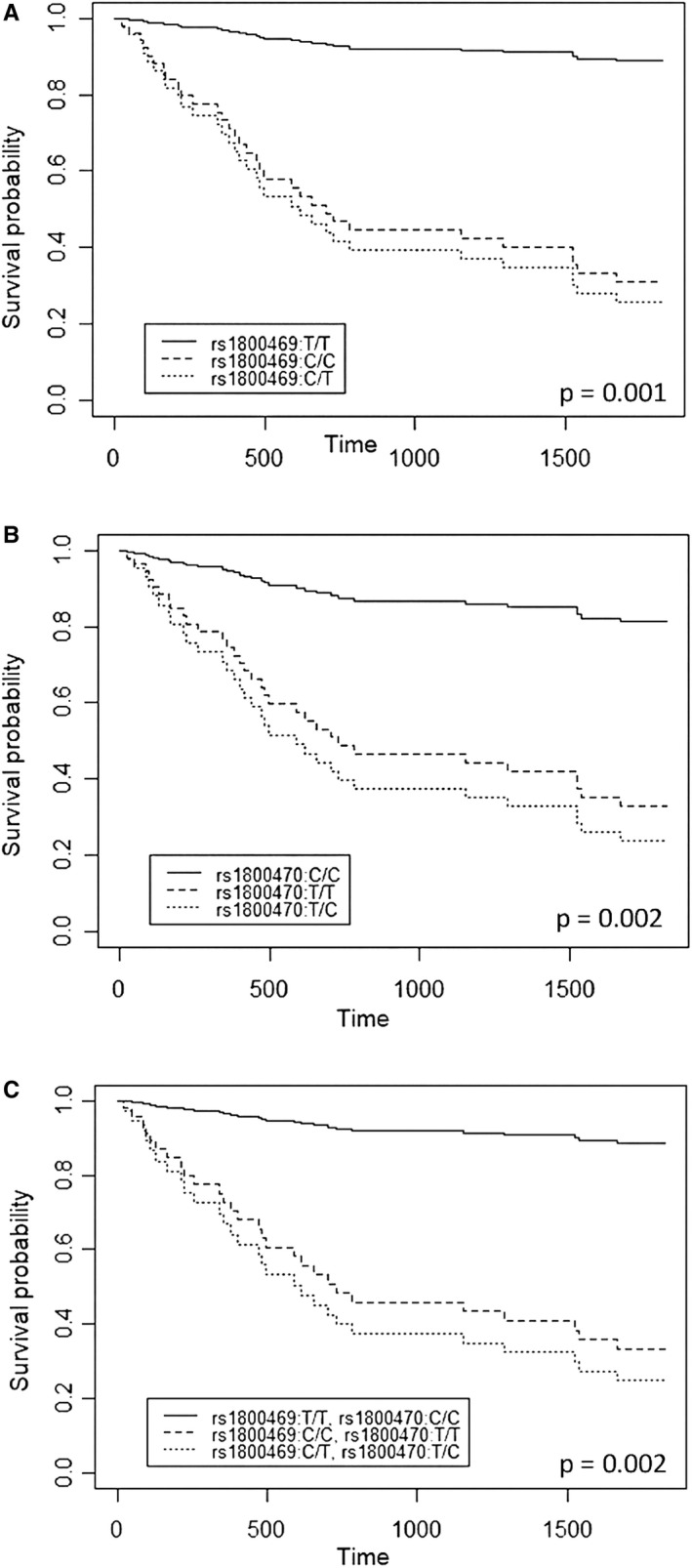
Survival curves (multivariate Cox regression analysis) for gastric cancer. SNP rs1800469 (A), rs1800470 (B), the haplotype thereof (C) adjusted by age (as a cofactor modulating survival rates) were considered in the analysis. Hazard ratio (HR) inter‐group comparison was assessed applying the Wald test. rs1800469‐C/T, rs1800469‐C/C, rs1800470‐T/C and rs1800470‐T/T genotypes, as well as the haplotypes rs1800469‐C/T + rs1800470‐T/C and rs1800469‐C/C + rs1800470‐T/T, are linked to a lower survival rate. *P* values shown in the boxes

To see whether TGFB1 polymorphisms had any effect on the patient's survival, we decided to test the SNPs rs1800469, rs1800470 (in linkage disequilibrium) and the previously mentioned CT risk haplotype (Figure 2). Multivariable Cox regression analysis indicates that, when adjusting by age (done in all comparisons carried out), SNPs rs1800469, rs1800470 and the combined haplotype exert an effect on the 5‐year survival. Thus, the Hazard Ratio (HR, the risk of death) for patients bearing the rs1800469‐C/C and rs1800469‐C/T genotypes was higher (HR = 9.9, 95%CI = 1.3‐75.7 and HR = 11.5, 95%CI = 1.5‐87.5, respectively) compared to patients bearing the rs1800469‐T/T genotype (Wald test *P*‐value = .027 and .019, respectively). As for the SNP rs1800470, HR of patients bearing the rs1800470‐T/T and rs1800470‐C/T genotypes was higher (HR = 5.3, 95%CI = 1.2‐23.9 and HR = 6.9, 95%CI = 1.6‐30.1, respectively) compared to patients bearing the rs1800470‐C/C genotype (Wald test *P*‐value = .029 and .011, respectively). Finally, if the haplotype is considered, the HR of patients bearing the combined haplotypes rs1800469‐C/C + rs1800470‐T/T (HR = 9.1, 95% CI = 1.2‐70.2, Wald test *P*‐value = .034) or rs1800469‐C/T + rs1800470‐T/C (HR = 11.5, 95% CI = 1.5‐88.5, Wald test *P*‐value = .019) is higher if compared to patients bearing the rs1800469‐T/T + rs1800470‐C/C haplotype. Additionally, for all the markers analysed, a 10‐year increase in the patient's age is associated with a hazard increase of about 50% (HR = 1.5, 95% CI (1.1‐1.9), Wald test *P*‐value = .006).

### 3.2 Cytokine studies

#### 3.2.1 TGF‐β1 production upon lymphocyte stimulation

**FIGURE 3:**
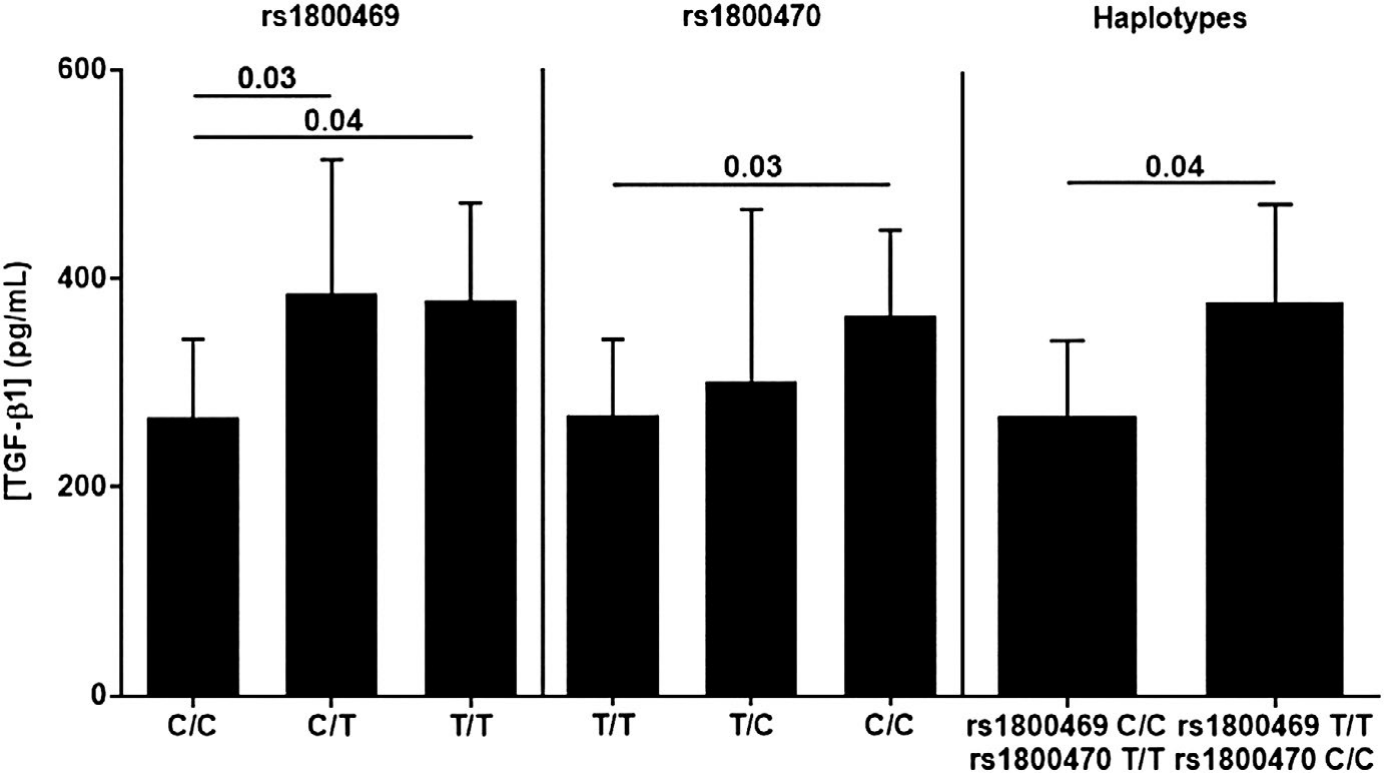
TGF‐β1 production and TGFB1 polymorphisms. PBMC from genotyped individuals was stimulated, and TGF‐β1 production was measured by ELISA. A significant difference in TGF‐β1 production was found between individuals rs1800469 C/C and C/T or T/T SNPs (C/C 268.4 pg/mL vs C/T 387.1 pg/mL, *P* = .04 or T/T 380.5 pg/mL, *P* = .03), as well as between rs1800470 T/T and C/C individuals (T/T 270.4 pg/mL vs C/C homozygous patients 365.8 pg/mL *P* = .03). When comparing Haplotypes, individuals bearing the rs1800469‐C/rs1800470‐T haplotype in homozygosis showed a significant lower expression of TGF‐β1 than individuals with the alternative haplotype rs1800468‐T/rs1800470‐C (281.6 pg/mL vs 380.5 pg/mL, *P* = .04)

Once the genetic profile of the TGFB1 gene was studied in our group of patients, we decided to test whether the polymorphisms analysed related to the amount of TGF‐β1 produced by PBMC upon stimulation with PMA and ionomycin (Figure 3), as described in Materials and Methods.

Although no significant differences were found in the case of SNPs rs1800468 and rs1800471 (probably because of the low frequency of these SNPs), the variants of rs1800469 and rs1800470 showed significant differences in TGF‐β1 synthesis, as previously described in other works. Regarding the rs1800469 polymorphism, PBMC from C/C patients produced lower amounts of TGF‐β1 (268.4 pg/mL ± 26.4 N = 8) than individuals with the C/T (387.1 pg/mL ± 74.2 N = 3, *P* = .04) or T/T variants (380.5 pg/mL ± 53.8 N = 3, *P* = .03). As for the rs1800470 polymorphism, PBMC from T/T homozygous individuals synthesized less TGF‐β1 (270.4 pg/mL ± 25.5 N = 8), than C/C homozygous patients (365.8 pg/mL ± 40.8 N = 4, *P* = .03).

Finally, PBMC from patients bearing the CT haplotype in homozygosis produced significantly lower amounts of TGF‐β1 (281.6 pg/mL ± 26.4 N = 7) than patients with the alternative haplotype rs1800469‐T/rs1800470‐C (TC) in homozygosis (380.5 pg/mL ± 53.8 N = 3, *P* = .04).

#### 3.2.2 TGF‐β1 plasma levels, disease progression and survival

Next to measuring TGF‐β1 production by PBMC from patients, we decided to measure the levels of the cytokine in the plasma of a further group of non‐genotyped patients with gastric adenocarcinoma (N = 30), to evaluate TGF‐β1 plasma levels as an independent prognostic marker.

**FIGURE 4:**
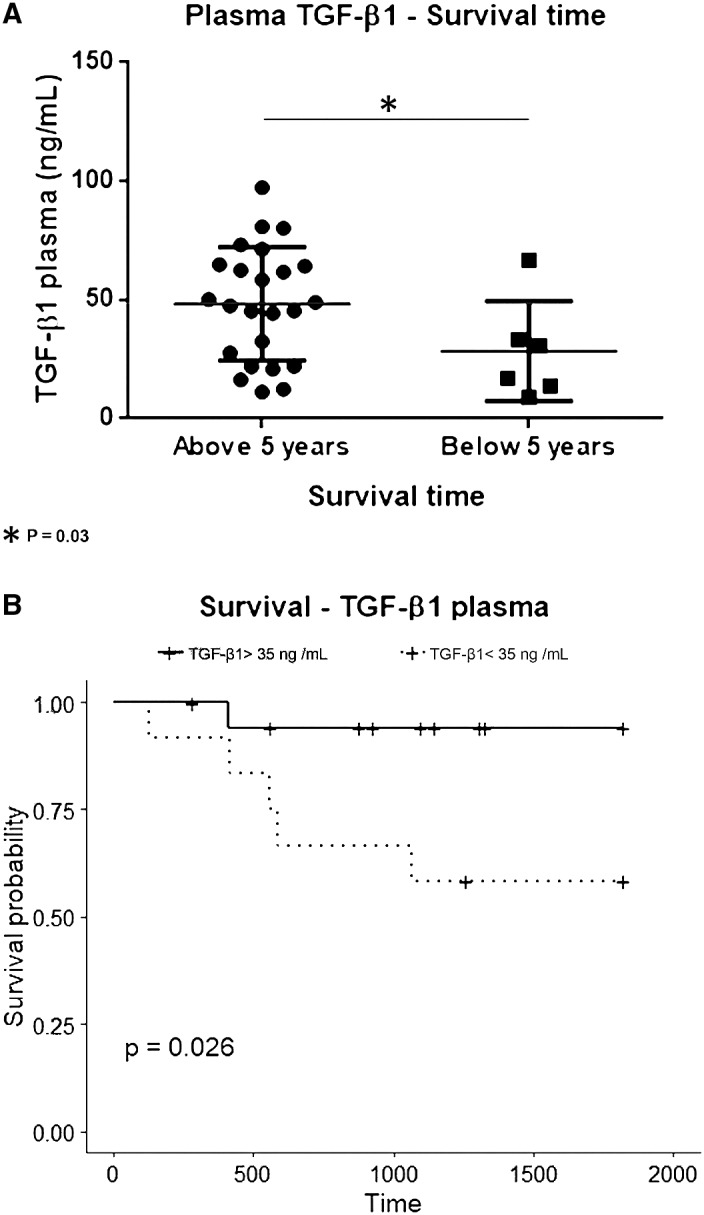
Plasma TGF‐β1 levels and survival rate. A, TGF‐β1 levels in plasma are lower in patients with a reduced survival time: Patients with a survival time below 5 y have a significant decrease of plasmatic levels of TGF‐β1 (28.3 ± 8.6 N = 6) compared to patients above that cut‐off 48.3 ± 4.9 N = 24, *P* = .03). B, Survival curves of patients with different levels of TGF‐β1 (TGF‐β1 > 35 ng/mL vs TGF‐β1 < 35 ng/mL). Log‐rank test detects a statistically significant difference in the survival rate of individuals with less than 35 ng/mL (58%) and patients with levels above this cut‐off (94%, log‐rank test *P* = .026). Kaplan‐Meier method was used to estimate the 5‐y survival function. Stage I, II and IV patients have been included in this analysis. No stage III patients were available

When patients were divided according to their TNM status, no differences in TGF‐β1 plasma levels were found between patients with non‐disseminated disease (stages I and II) and patients with disseminated disease (stage IV) (data not shown). However, if patients were classified according to their 5‐year survival, plasma TGF‐β1 was lower in patients below the 5‐year cut‐off (27.1 ng/mL, N = 9) compared to patients above it (49.2 ng/mL, N = 36, *P* = .03). This suggests that, regardless of the disease stage, patients with lower levels of TGF‐β1 have a faster and aggressive progression of the disease (Figure 4A). As TGFB1 polymorphisms were not carried out in this group of patients, their effect on the cytokine levels could not be assessed.

We then sought to determine a cut‐off point above which we could predict the outcome of a given patient and found that patients with less than 35 ng/mL of TGF‐β1 in plasma showed a significant lower survival rate (N = 12, 58%) than patients with levels above that cut‐off (N = 18, 94%, Log‐rank test *P* = .026) (Figure 4B).

These results must be taken with caution because they have been obtained certainly with a reduced number of patients and need to be confirmed in a larger group.

## 4 DISCUSSION

The quest for markers able to pinpoint gastric adenocarcinoma patients prone to disseminated metastases is relevant. For clinicians facing a newly diagnosed patient, the use of such tools will be advantageous when establishing a therapeutic approach. In this sense, the study of the TGFB1 SNPs presented may be of major interest in clinical practice.

### 4.1 Genetic studies

We found an association between rs1800471 polymorphisms and the development of gastric cancer. Particularly, the frequency of the G/C genotype is more than double in the group of patients with gastric cancer (20.7%) than in controls (8.4%; 1000 Genomes Database *P* = .027), suggesting that possessing the rs1800471‐G/C genotype increases the risk of developing gastric cancer. Moreover, the C allele of this SNP had been linked with a decreased expression of the cytokine TGF‐β1.^12^, ^13^ As other authors demonstrated,^23^, ^24^, ^25^ lack of TGF‐β1 signalling resulted in the development of much more aggressive tumours, either spontaneous or in presence of a carcinogen, suggesting that low TGF‐β1 levels, together with other exogenous factors, can facilitate the appearance of gastric tumours.

Some of the SNPs analysed are able to identify patients who will suffer disseminated disease (stage IV, according to TNM classification) during the progression of the disease as compared to those with early cancer or local dissemination (stages I, II and III, combined). Thus, the frequency of G/A genotype of the rs1800468 SNP, T/T genotype (rs1800469) and the ACTG combined haplotype (of the rs1800468, rs1800469, rs1800470 and rs1800471 SNPs, respectively) do significantly differ between both groups of patients (*P* = .04, .008 and .02, respectively). Remarkably, no T/T individuals were found in stage IV patients.

Previously published data in other types of tumours focused on comparing healthy controls with patients to use these polymorphisms as a diagnostic tool, but few works, especially in gastric cancer, examine TGFB1 as a prognosis tool within the stages of the disease.

Altogether, these results indicate that the genetic markers herein studied are involved in the progression to advanced stages of the disease, as occurs in other epithelial type malignancies (colorectal cancer).^25^, ^26^

Research on the relationship between the TGFB1 gene polymorphisms and the development of gastric cancer focused mainly on disease susceptibility in case‐control studies, comparing these SNP frequencies between a cohort of patients and a control group.^27^, ^28^ Normally, no group subdivisions were carried out according to the stage of the patients and, if they were, stage I and II patients were taken as a group and stage III or IV patients as another group. However, we believe that a division according to the absence (stage I, II and III) or presence (stage IV) of advanced disseminated disease is more sound clinically and in terms of prognosis,^29^ although it may substantially limit the number of patients available to carry out inter‐group comparisons.

As previously mentioned, these single base polymorphisms are located in key areas involved in the genetic regulation of this cytokine: two of them in the promoter region and the other two in exon 1, which encodes for the signal peptide of the protein.

Regarding the SNPs of the promoter region, several articles describe their interaction with different transcription factors that, in the regulation processes of this cytokine during tumorigenesis, would either favour or impede the union of certain activating transcription factors, such as CREB to rs1800468 or AP1 to rs1800469, respectively.^11^, ^15^ Similar findings have been reported in other clinical conditions such as asthma: variations in the promoter region regulate the binding of the Yin Yang1 transcription factor and therefore the amount of TGF‐β1 produced and the clinical evolution of the disease.^30^

Exon 1 of the TGFB1 gene encodes for the signal peptide of this protein and thus variations in the DNA sequence may involve changes in the secretion of this cytokine to the extracellular medium.^14^ Based on previously published data and our results, the significant increase in the frequency of the ACTG haplotype in stage IV patients implies that these individuals would be less able to release TGF‐β1 in the tumour environment. In fact, previous works describe that changes in the TGF‐β1 levels within the tumour are significant in early stages of the disease (types I and II patients), but the differences are lost if patients in more advanced stages of the pathology are considered.^31^

In conclusion, analysis of TGFB1 SNPs identified individuals at risk of developing gastric cancer, presenting with a more aggressive form of disease (stage IV) and reduced life expectancy.

#### 4.1.1 TGFB1 markers and survival

We describe a TGFB1 haplotype linked to life expectancy. Thus, survival of stage I, II and III patients bearing the haplotype rs1800469‐C/rs1800470‐T is significantly lower than that of patients bearing the alternative haplotype (Figure 3C).

A possible explanation is that in patients bearing this haplotype in homozygosis, the levels of TGF‐β1 synthesized, known to affect tumour progression, could be lower than in individuals with the alternative haplotype, who are thus protected against the progression of the tumour.

To the best of our knowledge, this is the first description of a TGFB1 extended haplotype affecting survival rates in a population of gastric cancer patients.

The presence of TGFB1 polymorphisms that condition changes in the expression of this cytokine can functionally mimic, although to a lesser extent, the clinical condition found in patients with inactivating mutations of the TGFBR2 receptor. Mutations in this receptor are very frequent in other types of neoplasms of the gastrointestinal tract^32^, ^33^ and also in other tumour types, such as glioblastoma,^34^ hepatocarcinoma^35^ and lung adenocarcinoma,^36^ where reduced expression of TGFBR2 increases tumour aggressiveness.

Therefore, a decrease in TGF‐β1 levels in early stages of the tumour would imply a defect in this signalling pathway, reducing the tumour suppressor effect of this cytokine and causing rapid tumour progression to more severe stages (growth of the tumour, invasion of adjacent lymph nodes and metastases).

### 4.2 Cytokine studies

By performing PBMC stimulation of carefully rs1800468‐A, rs1800469‐C and rs1800470‐T genotyped patients, we found that carriers of these polymorphisms were less able to produce TGF‐β1, in keeping with published data. It is then conceivable that this TGF‐β1 production may occur in the tumour environment in patients with the above‐mentioned alleles and the combined haplotype thereof (Figure 2).

These data lend further support to the notion that the disseminated disease‐linked genotype and haplotype leads to lower TGF‐β1 expression and concomitant faster progression of the disease; this haplotype is thus a genetic signature of patients at risk of developing a more severe form of the disease.

In line with the idea above, we went on to measure plasma TGF‐β1 levels (obtained before treatment and resection of the tumour) in a group of patients. Lower levels were found in patients with a survival time lower than 5 years (28.34 ng/mL ± 8.60, N = 6) compared to patients above this time point (48.34 ng/mL ± 4.89, N = 24, *P* = .03) (Figure 4A) and, in our hands, 35 ng/mL plasma TGF‐β1 is and adequate cut‐off point to assess survival of patients (Figure 4B).

Taken together, these data support the notion that TGF‐β1 acts as a tumour suppressor in this malignancy and that high levels of this cytokine limit the growth and aggressiveness of this tumour.

In summary, we report here that metastases‐prone gastric adenocarcinoma patients show higher frequency of TGFB1 SNPs associated with lower TFG‐β1 production. These SNPs could help identify patients at risk of developing advanced disseminated disease and lower life expectancy, requiring a more aggressive surgical and therapeutic approach early on. In addition, this finding could also explain the proposed dual role of this cytokine on tumour progression: in the initial stages of the disease, low amounts would render individuals more susceptible to an aggressive evolution of the disease. In keeping with this notion, we found that TFG‐β1 plasma levels were lower in patients with reduced life expectancy.

Therefore, we suggest the analysis of SNPs rs1800468, rs1800469 and rs1800470 and TGF‐β1 plasma levels, as markers to identify gastric cancer patients with poor prognosis.

#### 4.2.1 Human rights statement and informed consent

All procedures followed were in accordance with the ethical standards of the responsible committee on human experimentation (institutional and national) and with the Helsinki Declaration of 1964 and later versions. Informed consent to be included in the study, or the equivalent, was obtained from all patients.

#### 4.2.2 Gastric cancer susceptibility

Genotype rs1800471‐G/C was more frequent in patients with gastric adenocarcinoma (20.7%) compared to genetically related controls (8.4%, OR 2.84, *P* = .03).

Association of TGFB1 polymorphisms to the absence (stages I, II and III) or presence (stage IV) of metastasis in gastric adenocarcinoma patients: SNP rs1800468‐G/A showed a significant augment in patients with metastasis (type IV) compared to non‐metastatic ones (stage I, II and III, OR 3.71 *P* = .04). Remarkably, no rs1800469‐T/T individuals were found in stage IV patients, a genotype associated with a higher activity of the TGFB1 promoter, and either the codominant or recessive model showed a significant difference (*P* = .03 and .008, respectively) in the distribution of this SNP between metastatic and non‐metastatic patients.

## CONFLICT OF INTEREST

The authors confirm that there are no conflicts of interest.

## AUTHOR CONTRIBUTION

**Jose M. Martin‐Villa:** Conceptualization; writing‐original draft; formal analysis; writing‐review and editing; Supervision. **Ignacio Juarez:** Investigation; Methodology; Software; writing‐review and editing; writing‐original draft. **Christian Vaquero‐Yuste:** Investigation; Software. **Elisa Morales‐Lopez:** statistics analysis, writing; software. **Alberto Gutierrez:** Resources; Visualization. **Adela Lopez:** Resources; Visualization. **Inmaculada Lasa:** Resources; Visualization. **Remedios Gomez:** Resources; Visualization.

## Data Availability

The data that support the findings of this study are available from the corresponding author upon reasonable request.

## References

[1] Bray F , Ferlay J , Soerjomataram I , et al. Global cancer statistics 2018: GLOBOCAN estimates of incidence and mortality worldwide for 36 cancers in 185 countries. CA Cancer J Clin. 2018;68:394‐424. 30207593 10.3322/caac.21492

[2] Jin Z , Jiang W , Wang L . Biomarkers for gastric cancer: progression in early diagnosis and prognosis (Review). Oncol Lett. 2015;9:1502‐1508. 25788990 10.3892/ol.2015.2959 PMC4356326

[3] Wu H , Lin W , Tsai K . Advances in molecular biomarkers for gastric cancer: miRNAs as emerging novel cancer markers. Expert Rev Mol Med. 2014;16:e1. 24456939 10.1017/erm.2013.16 PMC3908627

[4] Baniak N , Senger J , Ahmed S , et al. Gastric biomarkers: a global review. World J Surg Oncol. 2016;14:212. 27514667 10.1186/s12957-016-0969-3 PMC4982433

[5] McLean MH , El‐Omar EM . Genetics of gastric cancer. Nat Rev Gastroenterol Hepatol. 2014;11:664‐674. 25134511 10.1038/nrgastro.2014.143

[6] Alexandrov LB , Nik‐Zainal S , Wedge DC , et al. Signatures of mutational processes in human cancer. Nature. 2013;500:415‐421. 23945592 10.1038/nature12477 PMC3776390

[7] Derynck R , Akhurst RJ , Balmain A . TGF‐beta signaling in tumor suppression and cancer progression. Nat Genet. 2001;29:117‐129. 11586292 10.1038/ng1001-117

[8] Travis MA , Sheppard D . TGF‐β activation and function in immunity. Annu Rev Immunol. 2014;32:51‐82. 24313777 10.1146/annurev-immunol-032713-120257 PMC4010192

[9] Lebrun J . The dual role of TGF in human cancer: from tumor suppression to cancer metastasis. Mol Biol. 2012;8:2019. 10.5402/2012/381428 PMC4899619 27340590

[10] Ranganathan P , Agrawal A , Bhushan R , et al. Expression profiling of genes regulated by TGF‐beta: differential regulation in normal and tumour cells. BMC Genom. 2007;8:98. 10.1186/1471-2164-8-98 PMC1858692 17425807

[11] Grainger DJ , Heathcote K , Chiano M , et al. Genetic control of the circulating concentration of transforming growth factor Type β1. Hum Mol Genet. 1999;8:93‐97. 9887336 10.1093/hmg/8.1.93

[12] Yokota M , Ichihara S , Lin TL , et al. Association of a T29–>C polymorphism of the transforming growth factor‐beta1 gene with genetic susceptibility to myocardial infarction in Japanese. Circulation. 2000;101:2783‐2787. 10859282 10.1161/01.cir.101.24.2783

[13] Nabrdalik K , Gumprecht J , Adamczyk P , et al. Association of rs1800471 polymorphism of TGFB1 gene with chronic kidney disease occurrence and progression and hypertension appearance. Arch Med Sci. 2013;9:230‐237. 23671432 10.5114/aoms.2013.34418 PMC3648826

[14] Yamada Y , Miyauchi A , Goto J , et al. Association of a polymorphism of the transforming growth factor‐beta1 gene with genetic susceptibility to osteoporosis in postmenopausal Japanese women. J Bone Miner Res. 1998;13:1569‐1576. 9783545 10.1359/jbmr.1998.13.10.1569

[15] Syrris P , Carter ND , Metcalfe JC , et al. Transforming growth factor‐beta1 gene polymorphisms and coronary artery disease. Clin Sci. 1998;95:659‐667. 10.1042/cs0950659 9831690

[16] Information National Center for Biotechnology , U. S. National Library of Medicine , 8600 Rockville Pike, MDB, USA 2. TGFB1 genetic polymorphisms and coronary heart disease risk: a meta‐analysis. Centre for Reviews and Dissemination (UK); 2012.

[17] Ferreira RR , Madeira FDS , Alves GF , et al. TGF‐β Polymorphisms are a risk factor for chagas disease. Dis Markers. 2018;2018:4579198. 29670670 10.1155/2018/4579198 PMC5835243

[18] Liao N , Zhao H , Chen M , Xie Z . Association between the TGF‐β1 polymorphisms and chronic obstructive pulmonary disease: a meta‐analysis. Biosci Rep. 2017;37:BSR20170747. 28784933 10.1042/BSR20170747 PMC5577172

[19] Osadnik T , Strzelczyk JK , Reguła R , et al. The relationships between polymorphisms in genes encoding the growth factors TGF‐β1, PDGFB, EGF, bFGF and VEGF‐A and the restenosis process in patients with stable coronary artery disease treated with bare metal stent. PLoS One. 2016;11:e0150500. 26930482 10.1371/journal.pone.0150500 PMC4773170

[20] Rodríguez Santiago JM , Sasako M , Osorio J . TNM‐7th edition 2009 (UICC/AJCC) and Japanese classification 2010 in gastric cancer. Towards simplicity and standardisation in the management of gastric cancer. Cir Esp. 2011;89:275‐281. 21256476 10.1016/j.ciresp.2010.10.011

[21] Auton A , Brooks LD , Durbin RM , et al. A global reference for human genetic variation. Nature. 2015;526:68‐74. 26432245 10.1038/nature15393 PMC4750478

[22] Solé X , Guinó E , Valls J , et al. SNPStats: a web tool for the analysis of association studies. Bioinformatics. 2006;22:1928‐1929. 16720584 10.1093/bioinformatics/btl268

[23] Park K , Kim SJ , Bang YJ , et al. Genetic changes in the transforming growth factor beta (TGF‐beta) type II receptor gene in human gastric cancer cells: correlation with sensitivity to growth inhibition by TGF‐beta. PNAS. 1994;91:8772‐8776. 8090721 10.1073/pnas.91.19.8772 PMC44688

[24] Ijichi H , Chytil A , Gorska AE , et al. Aggressive pancreatic ductal adenocarcinoma in mice caused by pancreas‐specific blockade of transforming growth factor‐beta signaling in cooperation with active Kras expression. Genes Dev. 2006;20:3147‐3160. 17114585 10.1101/gad.1475506 PMC1635149

[25] Muñoz NM , Upton M , Rojas A , et al. Transforming growth factor beta receptor type II inactivation induces the malignant transformation of intestinal neoplasms initiated by Apc mutation. Cancer Res. 2006;66:9837‐9844. 17047044 10.1158/0008-5472.CAN-06-0890

[26] Markowitz SD , Bertagnolli MM . Molecular origins of cancer: molecular basis of colorectal cancer. N Engl J Med. 2009;361:2449‐2460. 20018966 10.1056/NEJMra0804588 PMC2843693

[27] Jin G , Wang L , Chen W , et al. Variant alleles of TGFB1 and TGFBR2 are associated with a decreased risk of gastric cancer in a Chinese population. Int J Cancer. 2007;120:1330‐1335. 17187359 10.1002/ijc.22443

[28] Lin X , Li C , Shi Y , et al. Correlation of polymorphism of Nme1‐1465 T>C and TGFβ1‐509 T>C with genetic susceptibility of gastric carcinoma. Zhonghua Bing Li Xue Za Zhi. 2010;39:681‐685. 21176535

[29] Guan X , Zhao H , Niu J , et al. Polymorphisms of TGFB1 and VEGF genes and survival of patients with gastric cancer. J Exp Clin Cancer Res. 2009;28:94. 19566948 10.1186/1756-9966-28-94 PMC2717936

[30] Silverman ES , Palmer LJ , Subramaniam V , et al. Transforming growth factor‐β1promoter polymorphism C–509T is associated with asthma. Am J Respir Crit Care Med. 2004;169:214‐219. 14597484 10.1164/rccm.200307-973OC

[31] Choi YJ , Kim N , Shin A , et al. Influence of TGFB1 C‐509T polymorphism on gastric cancer risk associated with TGF‐β1 expression in the gastric mucosa. Gastric Cancer. 2015;18:526‐537. 25118995 10.1007/s10120-014-0412-9

[32] Biswas S , Trobridge P , Romero‐Gallo J , et al. Mutational inactivation of TGFBR2 in microsatellite unstable colon cancer arises from the cooperation of genomic instability and the clonal outgrowth of transforming growth factor beta resistant cells. Genes Chromosomes Cancer. 2008;47:95‐106. 17985359 10.1002/gcc.20511

[33] Xu Y , Pasche B . TGF‐beta signaling alterations and susceptibility to colorectal cancer. Hum Mol Genet. 2007;16(1):14. 10.1093/hmg/ddl486 PMC2637552 17613544

[34] Narushima Y , Kozuka‐Hata H , Koyama‐Nasu R , et al. Integrative network analysis combined with quantitative phosphoproteomics reveals transforming growth factor‐beta receptor type‐2 (TGFBR2) as a novel regulator of glioblastoma stem cell properties. Mol Cell Proteomics. 2016;15:1017‐1031. 26670566 10.1074/mcp.M115.049999 PMC4813685

[35] Mamiya T , Yamazaki K , Masugi Y , et al. Reduced transforming growth factor‐beta receptor II expression in hepatocellular carcinoma correlates with intrahepatic metastasis. Lab Invest. 2010;90:1339‐1345. 20531292 10.1038/labinvest.2010.105

[36] Xu J , Bao Y , Liu X , et al. Defective expression of transforming growth factor beta type II receptor (TGFBR2) in the large cell variant of non‐small cell lung carcinoma. Lung Cancer. 2007;58:36‐43. 17566598 10.1016/j.lungcan.2007.04.019

